# Evaluation of a point-of-care meter for measuring glucose concentrations in dairy calves: A diagnostic accuracy study

**DOI:** 10.3168/jdsc.2021-0190

**Published:** 2022-04-26

**Authors:** D.L. Renaud, K.S. Hare, K.M. Wood, M.A. Steele, M.C. Cantor

**Affiliations:** 1 Department of Population Medicine, University of Guelph, Guelph, ON, Canada, N1G 2W1; 2 Department of Animal Biosciences, University of Guelph, Guelph, ON, Canada N1G 2W1

## Abstract

The objective of this cross-sectional, diagnostic accuracy study was to validate a human blood glucose meter (Contour Next One Meter, Ascensia Diabetes Care) for accuracy and precision when measuring blood glucose, and for diagnostic accuracy for hypoglycemic status in dairy calves using whole blood and blood plasma. A total of 49 male dairy calves [body weight (BW): 46.3 ± 0.8 kg] had jugular catheters placed within 75 min after birth. Thereafter, blood was withdrawn from the catheter at specific time points (−10, 10, 20, 30, 45, 60, 90, 120, 180, 240, 360, 480, and 600 min) relative to the first and second colostrum feedings (2 h 15 min and 14 h 5 min postnatal; feeding rate: 7% of BW wt/wt). The reference standard method for plasma glucose concentration was determined colorimetrically and in duplicate using the glucose oxidase-peroxidase reaction. Data were assessed for agreement between the glucose meter and the reference standard using Lin's concordance correlation coefficients (CCC), coefficients of determination (precision), and Bland-Altman plots. In addition, a mixed linear regression model was built using the reference method as the outcome, with the glucose meter and repeated measures of time as the explanatory variables and calf as a random effect. The sensitivity (Se), specificity (Sp), and area under the curve (AUC) for the glucose meter using calf whole-blood and plasma were calculated at a threshold of <4.44 mmol/L to determine hypoglycemia. The precision (CCC = 0.95, R^2^ = 0.93) and accuracy (AUC = 0.98) of the glucose meter were very high when used on 1,303 blood plasma samples. Youden's index revealed a threshold of <4.45 mmol/L for the glucose meter when used with plasma, leading to Se of 94.2% and Sp of 91.9%, with 92.5% of samples being correctly classified, suggesting high diagnostic accuracy. When using whole blood, precision (CCC = 0.85 and R^2^ = 0.73) and accuracy (AUC = 0.92) were high when used on 476 samples. Youden's index revealed a threshold of <4.95 mmol/L for the glucose meter when used with whole calf blood, leading to Se of 95.6% and Sp of 80.3%, with 84.7% of samples being correctly classified, suggesting high diagnostic accuracy for use on farm. In summary, this glucose meter was validated for measuring calf blood glucose using both plasma and whole blood. This meter can measure glycemic status in calves and may be useful for clinical and on-farm use to make intervention decisions.

## Highlights

• We validated a human glucose meter for measuring blood glucose of neonatal calves using 1,303 samples of plasma and 476 samples of whole blood.
• For plasma, hypoglycemia was established at a threshold of <4.45 mmol/L (sensitivity, 94.2%; specificity, 91.2%).
• For whole blood, hypoglycemia was established at a threshold of 4.95 mmol/L (sensitivity, 95.6%; specificity of 80.3%).
• The meter can measure glycemic status in calves and may be useful for treatment decisions on farm.

## Graphical Abstract

**Figure:**
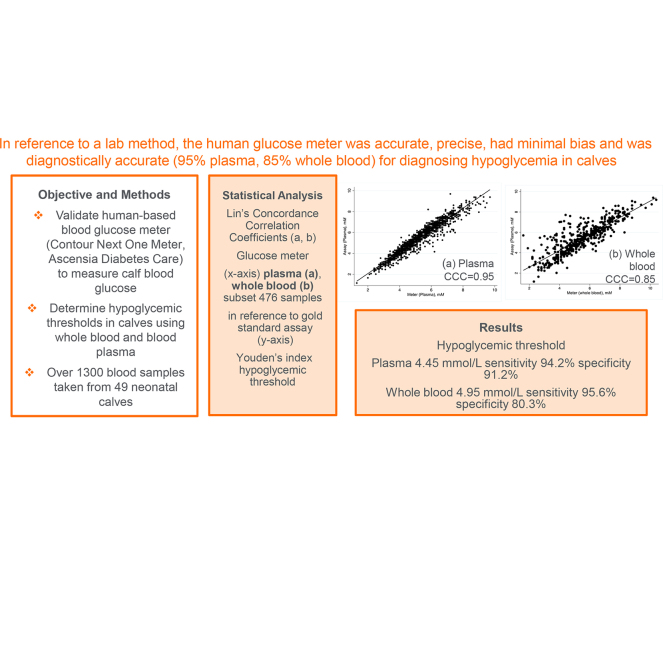
**Summary:** The objective of this study was to validate a blood glucose meter for measuring calf blood glucose and validate the diagnostic accuracy for hypoglycemic thresholds in dairy calves. A total of 49 male dairy calves had blood samples taken from jugular catheters at -10, 10, 20, 30, 45, 60, 90, 120, 180, 240, 360, 480, and 600 min relative to the first and second colostrum feeding. In reference to a laboratory method, the glucose meter was accurate, precise, and diagnostically accurate (95% plasma, 85% whole blood) for diagnosing hypoglycemia in calves.

Neonatal calves are born with limited body energy reserves (Okamoto et al., 1986; Quigley and Drewry, 1998) and a low circulating blood glucose concentration (e.g., 3 to 3.5 m*M*; Kirovski et al., 2011). This causes young calves to be susceptible to hypoglycemia during fasting or when blood nutrient availability changes, which is partly due to limited availability of nutrient reserves (Stämpfli and Oliver-Espinosa, 2014). During periods of disease, nutrient intake declines or nutrient demand increases to mount an immune response (Stämpfli and Oliver-Espinosa, 2014). This leads to hypoglycemia, which has been found in calves with diarrhea (Tennant et al., 1968; Lewis et al., 1975; Stämpfli and Oliver-Espinosa, 2014), sepsis, and endotoxemia (Ballou et al., 2011; Stämpfli and Oliver-Espinosa, 2014; Trefz et al., 2017). In addition to the association of hypoglycemia with morbidity in calves, hypoglycemic calves are also at an increased risk of mortality. For example, Trefz et al. (2016) performed a retrospective study using data from 10,060 hospitalized calves under 21 d of age and found that hypoglycemic calves were less likely to survive than normoglycemic calves. Furthermore, diarrheic hypoglycemic calves had shorter survival times than normoglycemic diarrheic calves, suggesting that low blood glucose levels may be a further risk factor for mortality (Tsukano et al., 2018). However, using gold standard laboratory assays to measure blood glucose takes time and equipment to process the sample and retrieve the calf blood plasma (Karapinar et al., 2020; Nishi et al., 2019). Calves that might be hypoglycemic need timely interventions, so it is important for a point-of-care test to be validated for use on farm (Trefz et al., 2016).

Cow-side blood glucose tests have been validated for mature cattle that produce results comparable to those of gold-standard laboratory testing (Lopes et al., 2019). However, these technologies are likely less effective for estimating blood glucose in calves due to the varying erythrocyte-to-plasma glucose ratio compared with that in mature cattle (Goodwin, 1956; Karapinar et al., 2020). Others have evaluated calf-side blood glucose tests (Nishi et al., 2019; Karapinar et al., 2020), but these point-of-care devices were either not accurate (Karapinar et al., 2020) or were not evaluated for hypoglycemic thresholds in calves (Nishi et al., 2019). It is essential to validate a blood glucose meter for both precision and accuracy (Costa et al., 2021) and to validate it for diagnostic accuracy to ensure that the device is measuring true blood glucose values to make appropriate intervention decisions regarding hypoglycemic status.

Because low blood glucose levels can have severe health consequences in calves, the ability to monitor and maintain normoglycemia would be beneficial for the wellbeing of these animals. Based on this, the objective of this cross-sectional diagnostic accuracy study was to validate a human-based point-of-care blood glucose meter (Contour Next One Meter, Ascensia Diabetes Care; reported accuracy of 97.4 to 100% and ±0.56 (SD) mmol/L for blood glucose concentrations at a threshold of 5.55 mmol/L) for accuracy and precision when measuring blood glucose status and to quantify the diagnostic accuracy of hypoglycemic status of these calves using whole blood and blood plasma. The Contour Next One Meter has an accuracy of 97% for blood glucose concentrations ≥5.55 mmol/L, and an accuracy of 100% compared with laboratory methods.

This study was reported using guidelines for essential items for reporting diagnostic accuracy studies (STARD 2015; Bossuyt et al., 2015). The study was conducted at a commercial dairy farm in southwestern Ontario, Canada, in accordance with the University of Guelph Animal Care Committee requirements (Animal Use Protocol #4126), as part of a larger study that evaluated how colostrum insulin concentrations affected blood metabolites and insulin concentrations, serum immunoglobulin G concentrations, and gastrointestinal development in calves. All singleton Holstein calves (n = 49; BW: 46.3 ± 0.8 kg) born between August 2019 and July 2020 to Holstein cows (median parity: 2; range: 1 to 6 lactations) were removed from their dams within 30 to 60 min of birth and enrolled. Calves were weighed (PS2000, Brecknell Scales, Avery-Weigh Tronix), vigorously towel-dried, and transferred to individual pens (1.22 m^2^) that were deeply bedded with wheat straw. We conducted a post hoc power analysis using methodology for diagnostic studies adapted from Serdar et al. (2021). At an α of 0.05, a maximum expected variation of 0.20 mmol/L in blood readings between hypoglycemic and normoglycemic calves, a maximum area under the curve (**AUC**) of 1, and an error of the glucose meter of 0.10, a sample size of 40 calves was required.

Within 75 min after birth, a 6.4-cm 16-gauge polyurethane intravenous catheter (SR*FF664, Terumo Surflash; Terumo Medical Canada Inc.) was placed aseptically within the jugular vein and extended with a 20-cm Luer-Lock extension line (Baxter 2N8378, Clearlink; Baxter International Inc.). Catheter patency was confirmed by withdrawing waste blood (5 mL) and flushing with 10 mL of heparinized (10 IU heparin/mL; C504710, Heparin, Fresenius Kabi Canada Ltd.) saline (0.9% NaCl; Baxter JB1324, Baxter International Inc.). Thereafter, blood was withdrawn from the catheter at specific time points (−10, 10, 20, 30, 45, 60, 90, 120, 180, 240, 360, 480, and 600 min) relative to the first and second colostrum feedings (2 h 15 min and 14 h 5 min postnatal; feeding rate: 7% of BW wt/wt). Waste blood (2 mL) was withdrawn and discarded before sample collection so that blood samples were not contaminated with heparin. Blood (14 to 18 mL per time point) was collected proportional to calf BW (total volume: 0.96% of BW, vol/wt), and an equivalent volume of 0.9% NaCl was reinfused to maintain fluid balance before flushing the catheter and extension line with 2 mL of heparinized saline, intended to prevent blood clots within the lines.

Blood was transferred to a Vacutainer coated with an anticoagulant (158 IU heparin; BD B366480, Becton Dickinson), and a competitive serine protease inhibitor (5 µg of aprotinin/mL of whole blood; Sigma-A1153, Millapore-Sigma) was immediately added to the blood. To validate the blood glucose meter with whole blood for use on farm, approximately 100 µL of whole blood was collected from a subset of 476 calves and immediately analyzed for real-time blood glucose concentration using the glucose meter and blood glucose test-strips (Type 7322; Contour Next, Ascensia Diabetes Care). We chose −10, 30, 60, 90, and 120 min relative to the first and second meals as our sampling subset to measure real-time postprandial blood glucose to ensure that calves did not risk becoming comatose from hypoglycemia following a colostrum meal supplemented with insulin (Pierce et al., 1964; Kirovski et al., 2008). We continued to monitor calves (n = 12) whose real-time blood glucose measurement dropped precipitously to <4.4 mmol/L past 120 min until their glucose concentrations recovered to normal levels. Immediately after the real-time blood glucose measurement was performed, Vacutainers were centrifuged (920 × *g*, 4°C, 25 min; TJ-6R Tabletop Centrifuge, Beckman Instruments) to separate plasma. Plasma was transferred in 3 aliquots to 1.5-mL microcentrifuge tubes, frozen, and stored at −20°C until analysis.

Plasma glucose concentration was determined in duplicate using the glucose oxidase/peroxidase reaction (P7119 and D3252; Sigma-Aldrich) and colorimetric detection (Trinder, 1969). Values were accepted when the coefficient of variation was <5% and inter- and intra-assay coefficients of variation were 1.41% and 1.64%, respectively. During analysis, 100 µL of plasma was simultaneously analyzed for plasma glucose concentration using the glucose meter and the blood glucose test-strips. Plasma glucose concentration measured by spectrophotometry was used as the reference method (or gold standard) to which we compared blood and plasma glucose concentrations measured by the glucose meter.

All statistical analyses were completed using Stata 17 (StataCorp LP). Data were imported from Excel (Windows 10, Microsoft Corp.) into Stata 17 and checked for completeness. To evaluate agreement between the reference method, and the glucose meter, Lin's concordance correlation coefficients (**CCC**) were calculated, where −1 = perfect disagreement and 1 = perfect agreement. In addition, to account for repeated measures through time, a repeated-measures mixed linear regression model was built, with the reference method as the outcome and the result of the glucose meter and time as explanatory variables and calf as a random effect. Using the method described by Selya et al. (2012), the coefficient of determination (**R^2^**) was calculated to determine the proportion of variance in the reference method explained by the result of the glucose meter.

Precision was defined by Lin's CCC and R^2^ and was categorized according to Hinkle (1988) as follows: 0.00–0.30 = negligible, 0.30–0.50 = low, 0.50–0.70 = moderate, 0.70–0.90 = high, and 0.90–1.00 = very high. We considered the glucose meter to be precise at measuring calf blood glucose when Lin's CCC was high and R^2^ was high. Bland-Altman plots (i.e., mean differences between the reference method and the glucose meter for calf plasma and whole blood) were generated to assess agreement and to measure for bias. We considered the data points to be free of bias in the Bland-Altman plot if 95% of the data points were contained within the limits of agreement (e.g., 95% CI).

Receiver operator characteristic (**ROC**) curves were developed using a threshold of <4.4 mmol/L for calf hypoglycemia (Trefz et al., 2016) to determine the sensitivity (**Se**) and specificity (**Sp**) of the glucose meter. The threshold for the glucose meter was calculated using the Youden's index, which determines the threshold that maximizes the Se and Sp of the meter. We considered the glucose meter to be accurate for measuring blood glucose status in calves if the 95% interval of agreement included zero for the mean bias from the Bland-Altman plots, and when the AUC from the ROC models had an AUC closer to 1. An ROC probability curve with an AUC closer to 1 indicated better classification of blood samples as hypoglycemic or not (Hoo et al., 2017).

The mean (± standard deviation) plasma glucose concentration determined using the reference method was 5.44 ± 1.50 mmol/L, whereas the mean plasma glucose concentration determined by the glucose meter was 5.29 ± 1.36 mmol/L. When using <4.4 mmol/L of glucose in plasma as a threshold for hypoglycemia (Trefz et al., 2016), 368 samples (27.2%) would have been classified as hypoglycemic. In a subset of time points, glucose concentration was measured in 476 whole-blood samples using the glucose meter. The mean glucose concentration determined by the meter on whole blood was 5.43 ± 1.61 mmol/L.

The CCC between plasma levels of glucose determined by the reference method and glucose meter was 0.95 (Figure 1a), indicating near perfect agreement. Accounting for the repeated measures over time, 93% of the variation in glucose concentration determined by the reference method was explained by the glucose meter (R^2^ = 0.93). Thus, the glucose meter was very precise for measuring plasma blood glucose concentrations in calves. A Bland-Altman plot (Figure 2a) revealed the mean bias was +0.22 ± 0.41 mmol/L, with 95% of the data points falling within the 95% limits of agreement (−0.58 to 1.02 mmol/L), indicating that the glucose meter had no bias compared with the reference method. The ROC curve for these data had an AUC of 0.98 (95% CI: 0.97 to 0.99). Thus, the glucose meter was very accurate for measuring plasma blood glucose in calves compared with the reference method. Based on Youden's index, we determined that the optimal threshold for hypoglycemia, defined as a plasma glucose of <4.4 mmol/L using the reference method, was 4.45 mmol/L when using plasma with the glucose meter. At this threshold, the Se and Sp were determined to be 94.2% and 91.9%, respectively, with 92.5% of samples being correctly classified, suggesting that the glucose meter was diagnostically accurate when using plasma glucose of calves.

**Figure 1:**
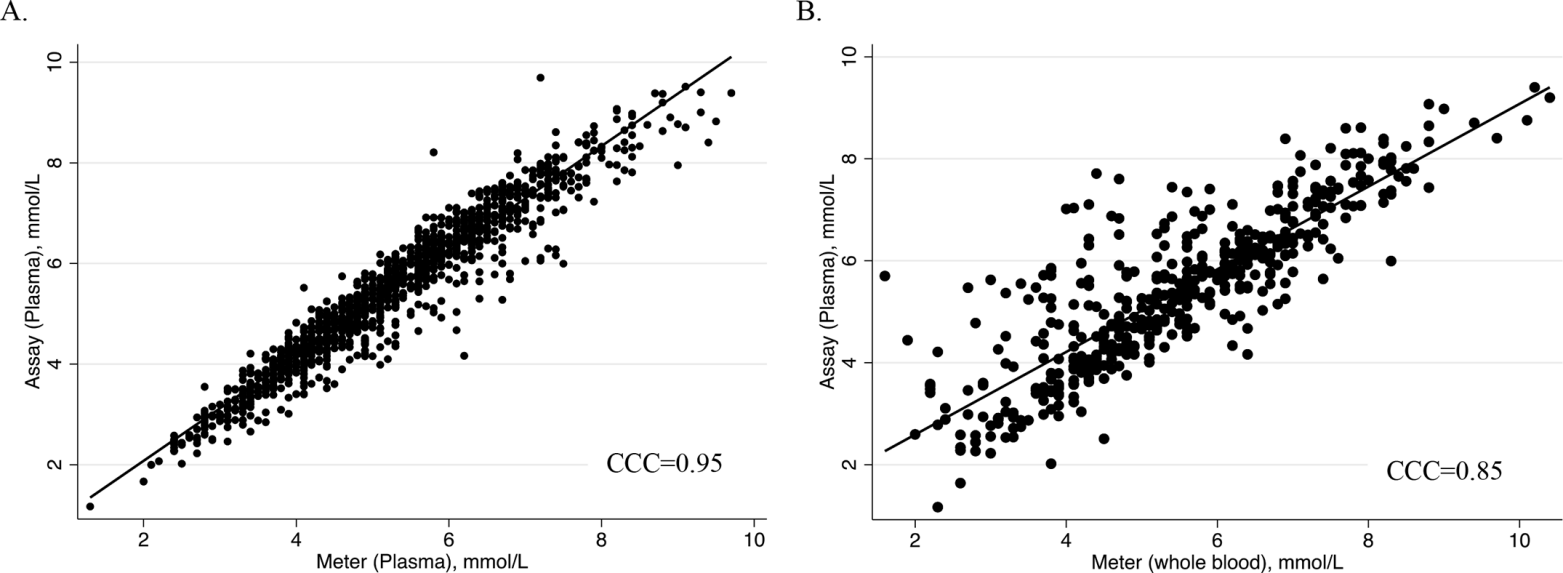
(A) Lin's concordance correlation coefficient (CCC) relationship between blood plasma glucose concentration as measured by glucose meter (Contour Next One Meter, Ascensia Diabetes Care) and by enzymatic spectrophotometry for 1,303 plasma samples taken from neonatal Holstein bulls (n = 49) at specific time points (−10, 10, 20, 30, 45, 60, 90, 120, 180, 240, 360, 480, and 600 min) relative to the first and second colostrum feeding (2 h 15 min and 14 h 5 min postnatal; feeding rate: 7% of BW). (B) Relationship between calf whole-blood glucose concentration as measured by glucose meter and plasma glucose concentration as measured by enzymatic spectrophotometry (assay) in a subset of 476 samples.

**Figure 2:**
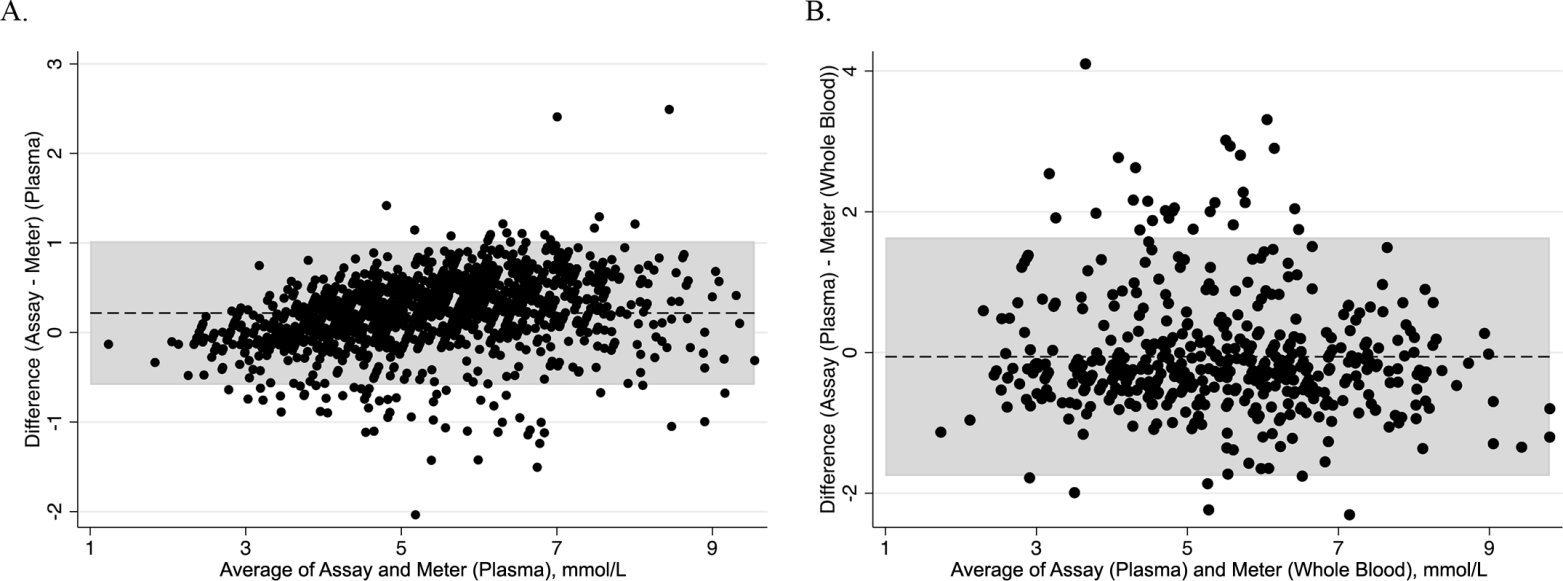
Mean difference (gray box = ±1.96 SD of the mean difference) measures of agreement (Bland-Altman) for calf blood glucose concentrations measured in plasma by enzymatic spectrophotometry (assay) or using the glucose meter (Contour Next One Meter, Ascensia Diabetes Care) in 1,303 samples taken from neonatal Holstein bulls (n = 49) at specific time points (−10, 10, 20, 30, 45, 60, 90, 120, 180, 240, 360, 480, and 600 min) relative to the first and second colostrum feeding (2 h 15 min and 14 h 5 min postnatal; feeding rate: 7% of BW). (A) Mean differences of 1,303 samples of calf blood plasma glucose concentration (reference method − glucose meter); (B) mean differences of 476 samples for blood glucose concentration (reference method − calf whole-blood glucose meter).

For the subset of the samples where calf whole blood was used, the CCC between plasma glucose concentration determined by the reference method and whole-blood concentrations of glucose determined by the meter was 0.85 (Figure 1b), indicating high agreement. In the repeated-measures linear regression model, the R^2^ was 0.73. Thus, the glucose meter was precise for measuring blood glucose in calves compared with the reference method. A Bland-Altman plot (Figure 2b) found that the mean bias was −0.06 ± 0.86 mmol/L, with 93.1% of data points falling within 95% limits of agreement (−1.75 to 1.64 mmol/L), indicating a minor bias to overestimate blood glucose compared with the reference method. The ROC curve for these data had an AUC of 0.92 (95% CI: 0.90 to 0.95), suggesting near perfect classification of the samples as hypoglycemic or not. Thus, the glucose meter was accurate when using whole blood compared with the reference method. A threshold for hypoglycemia was determined to be 4.95 mmol/L when using the glucose meter on whole blood, with a sensitivity of 95.6% and specificity of 80.3% at this threshold. In addition, 84.7% of samples were correctly classified using this threshold, suggesting that this glucose meter may be a useful tool for assessing for hypoglycemic status in calves on farm.

Although the glucose meter used in this study was originally validated to measure blood glucose concentrations in humans, it was accurate and precise and shows promise for identifying hypoglycemic status in calves when measured against the reference standard. Specifically, when glucose was measured directly on calf plasma, the glucose meter was very highly precise, very highly accurate, and had a reasonably high Se and Sp for identifying calves with hypoglycemia compared with the reference standard. Thus, we suggest that the glucose meter was validated and nearly representative of the gold standard for measuring blood glucose in calves when applied directly to plasma. When the glucose meter was used on calf whole blood compared with the reference standard using plasma, it was precise and accurate but had lower diagnostic accuracy, with a greater rate of false positives when identifying calves with hypoglycemia. However, we still suggest that the glucose meter could be a useful diagnostic tool to assess for hypoglycemic status in calves using whole blood on farm, although it should not replace laboratory reference methods.

Clinically, this point-of-care meter would be used to determine whether calves had hypoglycemia. Using the threshold identified by Trefz et al. (2016) for hypoglycemia in calves, the glucose meter in this study was highly accurate in both plasma and whole blood as indicated by the high AUC values. As identifying and treating hypoglycemia could help to reduce mortality associated with diarrhea and sepsis (Trefz et al., 2017; Tsukano et al., 2018), clinicians operating in farm settings could use this meter to identify hypoglycemic calves and create an intravenous solution with supplemental glucose (Constable et al., 2021). Furthermore, the use of this glucose meter could be clinically relevant for veterinarians making on-farm decisions; calves identified with hypoglycemia before surgery have poor prognosis and low survival rates due to high postoperative complications (Lausch et al., 2021). In addition, we tested the blood glucose meter following 2 meals 12 h apart; therefore, our results encompass the variation across a wide range of postprandial glycemic values. Thus, we suggest that the use of a glucose meter on farm to manage calves with diseases may improve decision-making regarding blood glucose treatments, irrespective of the interval from their prior meal. Future research should continue to investigate whether other diseases, such as bovine respiratory disease, are associated with glucose status in calves to add to the diagnostic value of this glucose meter in the field.

Other point-of-care glucose meters have been previously evaluated to measure blood glucose in calves. Specifically, the Freestyle Optium Neo H (Abbott Diabetes Care Inc.) was evaluated by Karapinar et al. (2020), and the Precision Xceed and i-STAT meters (Abbott Diabetes Care Ltd.) were validated by Nishi et al. (2019). Karapinar et al. (2020) also calculated the Se and Sp of diagnosing hypoglycemia in sick calves using a threshold of <4.0 mmol/L; they observed test performance similar to our findings. The similar performance is not unexpected because these meters are designed to provide accurate readings of circulating glucose in humans. However, according Karapinar et al. (2020), the glucose meter evaluated in their study was only useful for indicating calf hypoglycemic status; the meter had precision, with high correlations between the reference method and the glucose meter, but there was substantial proportional bias between the reference method and the glucose meter. Therefore, we conclude that the glucose meter evaluated by Karapinar et al. (2020) had poor agreement, proportional bias, and low accuracy, but was precise for measuring blood glucose in calves. This is different from our findings: we found high precision and accuracy, as well as diagnostic accuracy of the glucose meter evaluated in our study. Moreover, the glucose meters evaluated by Nishi et al. (2019) were validated as both accurate and precise for evaluating blood glucose status in calves, which agrees with our findings. However, caution is warranted because Nishi et al. (2019) removed 11% (11/96) of their samples due to high readings, which may have biased the strength of the relationship observed between these glucose meters and the reference method. Additionally, unlike our study, Nishi et al. (2019) did not evaluate for a hypoglycemic threshold in calves. However, performance of the meters between our study and Nishi et al. (2019) were similar, where the meters showed a high level of correlation with the reference method, suggesting precision, and a small number of data points outside the limits of agreement in the Bland-Altman plot, suggesting accuracy. Thus, we suggest that the glucose meter used in our study was validated to evaluate blood glucose and hypoglycemic status in calves.

Some limitations should be considered when interpreting the results of this study. All calves within this study were healthy, which could affect the results when the meter is used with clinically abnormal animals. For example, the ROC curves were developed using a threshold of <4.4 mmol/L for calf hypoglycemia (Trefz et al., 2016) to determine the Se and Sp of the glucose meter in this study. However, the hypoglycemic threshold reported by Trefz et al. (2016) was established for calves hospitalized for diarrhea. It is possible that measures in hydrated calves do not directly correspond with measures in diarrheic calves. However, as reported by Karapinar et al. (2020), glucose concentrations were not normally distributed for hospitalized calves (the median glucose concentration reported was 4.72 mmol/L), making comparisons with our findings difficult. Thus, we enrolled young healthy calves at the same age and followed them for multiple time points relative to feeding to ensure we validated the glucose meter without introducing the confounder of disease status. This was important because other studies that used hospitalized, diseased calves observed that blood glucose readings varied widely (Karapinar et al., 2020), that extremely low blood glucose status was associated with an increased risk of mortality (Trefz et al., 2016), and that low blood glucose status in calves was associated with poor surgical outcomes (Lausch et al., 2021). We ensured a sample size large enough to capture calves positive and negative for hypoglycemia that were not presenting clinical signs of disease. Furthermore, we acknowledge that postnatal calves have an immature gastrointestinal tract, and that postnatal calves depend on the first colostrum meal to stimulate enzyme production and increase glucose kinetics in the calf (Hammon et al., 2013). However, it is important to note that the mean blood glucose status of calves in our study was similar to that of a previous validation study, which used larger calves (>100 kg), suggesting that the mean glucose concentration of the younger calves used in our study was comparable to that of older calves (Nishi et al., 2019). In addition, the intra-assay coefficient of the glucose meter was not calculated, thus we did not evaluate the repeatability of this device. However, we did validate this device for both precision and accuracy, suggesting that the repeatability of the device is likely high. Thus, we recommend that calf whole-blood samples should not replace the reference gold standard, but that the acceptable Se and Sp of this meter makes is a useful diagnostic tool to make intervention decisions on farm.

In conclusion, this glucose meter was validated for measuring blood glucose status in calves; it was accurate and precise when used on calf blood plasma and whole blood. The diagnostic accuracy of this glucose meter to measure hypoglycemic status in calves using whole blood may aid clinicians and producers to make better treatment decisions, and using calf blood plasma may aid researchers seeking alternative diagnostic methods. Future research should investigate additional clinical applications of glucose status in calves.
